# Regression analysis for predicting the elasticity of liquid crystal elastomers

**DOI:** 10.1038/s41598-022-23897-0

**Published:** 2022-11-17

**Authors:** Hideo Doi, Kazuaki Z. Takahashi, Haruka Yasuoka, Jun-ichi Fukuda, Takeshi Aoyagi

**Affiliations:** 1 National Institute of Advanced Industrial Science and Technology (AIST), Research Center for Computational Design of Advanced Functional Materials, Central 2, 1-1-1 Umezono, Tsukuba, Ibaraki 305-8568 Japan; 2 Research Association of High-Throughput Design and Development for Advanced Functional Materials, Central 2, 1-1-1 Umezono, Tsukuba, Ibaraki 305-8568 Japan; 3 Panasonic Corporation, 3-1-1 Yagumo-naka-machi, Moriguchi, Osaka 570-8501 Japan; 4 Department of Physics, Faculty of Science, Kyushu University, 744 Motooka, Nishi-ku, Fukuoka, Fukuoka 819-0395 Japan

**Keywords:** Materials science, Soft materials, Liquid crystals, Polymers

## Abstract

It is highly desirable but difficult to understand how microscopic molecular details influence the macroscopic material properties, especially for soft materials with complex molecular architectures. In this study we focus on liquid crystal elastomers (LCEs) and aim at identifying the design variables of their molecular architectures that govern their macroscopic deformations. We apply the regression analysis using machine learning (ML) to a database containing the results of coarse grained molecular dynamics simulations of LCEs with various molecular architectures. The predictive performance of a surrogate model generated by the regression analysis is also tested. The database contains design variables for LCE molecular architectures, system and simulation conditions, and stress–strain curves for each LCE molecular system. Regression analysis is applied using the stress–strain curves as objective variables and the other factors as explanatory variables. The results reveal several descriptors governing the stress–strain curves. To test the predictive performance of the surrogate model, stress–strain curves are predicted for LCE molecular architectures that were not used in the ML scheme. The predicted curves capture the characteristics of the results obtained from molecular dynamics simulations. Therefore, the ML scheme has great potential to accelerate LCE material exploration by detecting the key design variables in the molecular architecture and predicting the LCE deformations.

## Introduction

Liquid crystal elastomers (LCEs) are a relatively new class of materials that display soft elasticity, that is, macroscopic reversible deformation has little resistance^1–6^. Soft elasticity can be achieved using a variety of external stimuli, such as stretching, thermal fields^7^, magnetic or electric fields^8–10^, and light exposure^11–16^. Furthermore, soft and light LCEs exhibit relatively fast and accurate reactions to these external stimuli. Therefore, LCEs are candidate materials for soft actuators^17–21^, and their mechanical properties have been extensively studied^22–25^. The mechanism of soft elasticity is closely related to the dynamics of mesogenic units embedded in polymer chains, and experiments have shown that a unidirectionally oriented polymer network with relatively low crosslink density is important for the realization of soft elasticity^6,26,27^. To clarify the coupling dynamics of mesogens and polymeric chains, theoretical studies have focused on the microscopic behavior of mesogens through molecular simulations^28–36^. These studies indicate the great potential of molecular simulations to uncover the mechanism of soft elasticity. Our previous study on the effect of LCE molecular architectures on microscopic dynamics under uniaxial elongation demonstrated that side-chain-type LCEs, in which mesogens are embedded in the side chain, have a different mesogen rotation mechanism from main-chain-type LCEs, in which mesogens are embedded in the main chain^34^. Furthermore, we found a systematic and robust trade-off between the stress and strain ranges in soft elasticity^35^, indicating that the optimal set of output power and amount of deformation for LCE can be selected by tuning the crosslink density. These are clear examples of how detailed information on the molecular architecture can be used to elucidate more realistic behavior in LCE molecular systems. However, vast variety of the molecular architecture often make it difficult to understand the relationship between the microscopic characteristics and macroscopic properties of materials. This is especially true in the case of functional polymeric materials such as LCEs. That is, the large number of variables that characterize the molecular architecture prevents the determination of which variables are important for the desired macroscopic properties. This difficulty arises in both experiments and simulations. In fact, despite the many possibilities for the molecular architecture of LCEs, experiments have employed only a few molecular structures that conform to some established synthetic methods^26,27,37^. In other words, the complexity of the material itself inhibits material development.

The microscopic characteristics of matter are expected to be closely related to its macroscopic physical properties^38–45^. However, finding a universal relationship remains a challenging task, with the exception of some successful examples limited to specific categories of phenomena or materials^38–40^. Microscopic characteristics are always complex, covering the chemistry, geometry, and dynamics of atoms and molecules. By contrast, macroscopic properties are not always sensitive to microscopic characteristics^46–48^. Moreover, the sensitivity of macroscopic properties to microscopic characteristics is material-dependent. Thus, it is often more efficient to focus exclusively on the details of the relationship between microscopic characteristics and macroscopic properties for a specific material, despite the potential significance of establishing universality across materials. For complex materials such as functional polymers, the details of the relationship remain difficult to investigate even when the class of the target material is fixed. This is because these materials have a number of microscopic parameters that constitute the design variables of the molecular architectures^49–51^. Complex information with many microscopic parameters makes it difficult to detect the major factors that characterize the macroscopic physical properties. Quantitative structure–property relationship (QSPR) analysis is a promising approach for overcoming this type of complexity. QSPRs have great potential to reveal the correlations between an objective variable (*i.e.*, physical property) and the large number of combinations of descriptors (*i.e.*, microscopic parameters), which are almost incomprehensible to humans^41–45,52–55^. Recently, the above characteristic of QSPRs has been enhanced by machine learning (ML) techniques and applied to materials science^56–59^. Many studies have suggested the use of specific physical properties for objective variables^56–61^, and several results have been reported for polymer elasticity^62–64^.

In this study, an ML-based QSPR approach is employed to identify the microscopic characteristics that govern the macroscopic deformation of LCEs. To identify the influential design variables, regression analysis using ML is performed on a database containing the results of coarse-grained molecular dynamics simulations of LCEs with various molecular architectures. The predictive performance of surrogate model is also tested using regression analysis. The LCE database contains design variables for LCE molecular architectures, system and simulation conditions, and stress–strain curves calculated for each LCE molecular system. In this study, data sets on 140 different LCE molecules are selected from the database. In addition, 12 molecules are randomly selected and excluded to test the predictive performance of surrogate model, with the remaining 128 molecules used in the regression analysis. Regression analysis is performed with the stress–strain curve as the objective variable and the other factors as explanatory variables. To test the predictive performance of the surrogate model, the stress–strain curves are predicted using ML results for the 12 LCE molecules that were excluded prior to regression.

## Results

### Regression analysis

**Figure 1:**
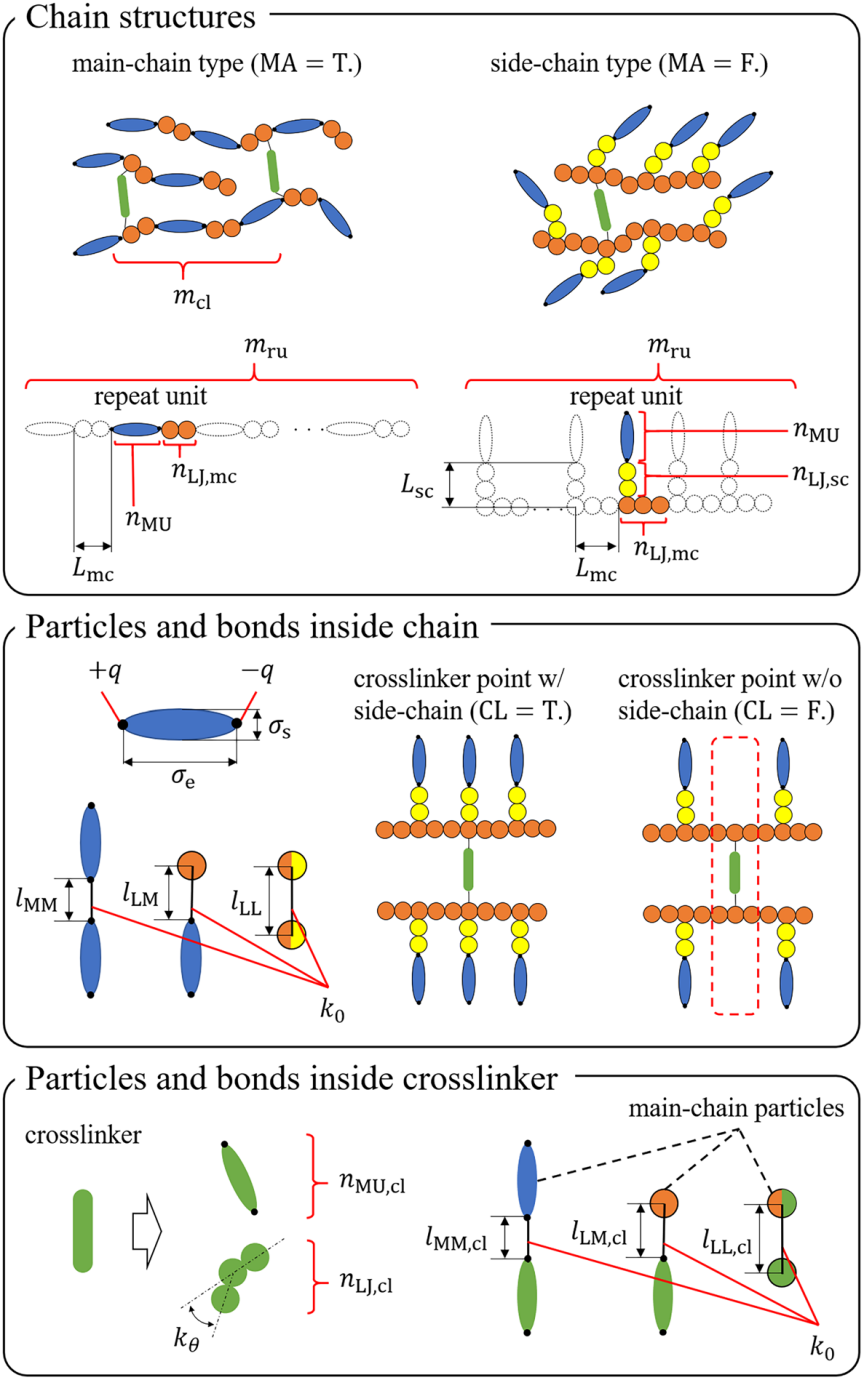
Schematic illustration of the design variables for LCE molecular architectures. Mesogenic units in chains are marked as blue ellipsoids, Lennard–Jones (LJ) particles in main chains are marked as orange circles, LJ particles in side chains are marked as yellow circles, mesogenic units in crosslinkers are marked as green ellipsoids, and LJ particles in crosslinkers are marked as green circles.

The mechanical properties of LCEs are expected to be related to the details of the molecular architectures, such as the difference between the main- and side-chain types, the type and density of the crosslinking agent, and the shape and density of the mesogens. Thus, in this study, a coarse-grained LCE model that can express the details of the molecular architectures is employed. A typical molecular architecture consists of soft-core Gay–Berne (SCGB) ellipsoidal particles^65^, Lennard–Jones (LJ) spherical particles, and harmonic bonds among particles (for details, see Ref.^34^). This model makes it possible to build numerous molecular architectures for LCEs by setting the 20 design variables shown in Fig. 1 and Table 1. A total of 220 main- and side-chain LCE molecules are modeled, corresponding to those considered in previous studies^34,35^. Note that the parameters for non-bonded interactions among SCGB and LJ particles, except the point charge *q*, are fixed to the same values as in Refs. 34 and 35, so they are not shown in Fig. 1 and Table 1.

**Table 1:** Design variables for LCE molecules. Variables that depend on some of the design variables are also displayed. The value “null” indicates that the variable does not exist in the molecular architecture.

Variable	Type	Description	Value(s)
MA	Logical	Main- or side-chain type	True (= main), False (= side)
$m_{\mathrm{ru}}$	Integer	Number of repeat units in the chain	30
$n_{\mathrm{MU}}$	Integer	Number of mesogenic units per repeat unit	1
$n_{\mathrm{LJ,mc}}$	Integer	Number of LJ units in the main chain of repeat unit	Null, 1, 2, 3
$n_{\mathrm{LJ,sc}}$	Integer	Number of LJ units in the side chain of repeat unit	Null, 1, 2, 3
*q*	Real	Point charge on the mesogenic unit	Null, 0.3
$\sigma _{\mathrm{e}}$	Real	Length of the long axis of mesogenic unit	3.0, 3.2
$\sigma _{\mathrm{s}}$	Real	Length of the short axis of mesogenic unit	1.0
$l_{\mathrm{MM}}$	Real	Length of the covalent bond between mesogenic units	Null, 0.15, 1, 1.2, 1.4, 1.6, 1.8, 2.0, 3.0
$l_{\mathrm{LL}}$	Real	Length of the covalent bond between LJ particles	Null, 1.0
$l_{\mathrm{LM}}$	Real	Length of the covalent bond between LJ particle and mesogenic unit	Null, 0.5
$k_0$	Real	Spring constant of the covalent bond	100.0, 500.0
CL	Logical	Presence or absence of the crosslinker point with the side chain	True (= present), False (= absent)
$m_{\mathrm{cl}}$	Integer	Number of crosslinkers in the chain	2, 3, 4, 6, 8
$n_{\mathrm{MU,cl}}$	Integer	Number of mesogenic units in the crosslinker	Null, 1
$n_{\mathrm{LJ,cl}}$	Integer	Number of LJ units in the crosslinker	Null, 1, 2, 3, 4, 5, 6
$k_{\theta }$	Real	Angle spring constant of the LJ crosslinker	Null, 10.0, 1000.0
$l_{\mathrm{MM, cl}}$	Real	Length of the covalent bond between mesogenic units in the main chain and mesogenic units in the crosslinker	Null, 0.0, 0.15, 0.5, 1.0
$l_{\mathrm{LL, cl}}$	Real	Length of the covalent bond between LJ particles in the main chain and crosslinker	Null, 1.0
$l_{\mathrm{LM, cl}}$	Real	Length of the covalent bond between LJ particle in the main chain and mesogenic unit in the crosslinker	Null, 0.15, 0.5
Dependent variable	Type	Description	
$r_{\mathrm{asp}}$	Real	$\sigma _{\mathrm{e}}/\sigma _{\mathrm{s}}$	
$L_{\mathrm{mc}}+L_{\mathrm{sc}}$	Real	$(n_{\mathrm{LJ,mc}} - 1) l_{\mathrm{LL}} + (n_{\mathrm{MU}} + 1)l_{\mathrm{LM}} + n_{\mathrm{MU}} l_{\mathrm{MM}}$ (for MA = True), $(n_{\mathrm{LJ,mc}}+n_{\mathrm{LJ,sc}})l_{\mathrm{LL}} + n_{\mathrm{MU}}(l_{\mathrm{LM}}+l_{\mathrm{MM}})$ (for MA = False)	

Molecular dynamics (MD) uniaxial elongation simulations yielded six stress-strain curves per LCE molecule (see Methods for details). Here, we present descriptors of the molecular system and elongation simulation conditions for regression analysis, along with specific set values. The number of chains $N_{\mathrm{ch}}$ for each system was fixed to the specific value shown in Table 2. The number of mesogenic units $N_{\mathrm{MU}}$ was $m_{\mathrm{ru}} \times n_{\mathrm{MU}} \times N_{\mathrm{ch}}$. The number of solvent SCGB particles $N_{\mathrm{solv}}$ was set to $N_{\mathrm{MU}}$ or multiples thereof. The number of mesogenic units in crosslinkers $N_{\mathrm{MU,cl}}$ was $m_{\mathrm{cl}} \times n_{\mathrm{MU,cl}} \times N_{\mathrm{ch}}$. The total number of SCGB particles $N_{\mathrm{SCGB}}$ was $N_{\mathrm{MU}}+N_{\mathrm{MU,cl}}+N_{\mathrm{solv}}$. The number of LJ particles in chains $N_{\mathrm{LJ, ch}}$ was $m_{\mathrm{ru}} \times (n_{\mathrm{LJ,mc}}+n_{\mathrm{LJ,sc}}) \times N_{\mathrm{ch}}$. The number of LJ particles in crosslinkers $N_{\mathrm{LJ, cl}}$ was $m_{\mathrm{cl}} \times n_{\mathrm{LJ,cl}} \times N_{\mathrm{ch}}$. The total number of LJ particles $N_{\mathrm{LJ}}$ was $N_{\mathrm{LJ, ch}}+N_{\mathrm{LJ,cl}}$. The total number of crosslinkers $N_{\mathrm{cl}}$ was $m_{\mathrm{cl}} \times N_{\mathrm{ch}}$. The chain rate $r_{\mathrm{ch}}$ was determined by $N_{\mathrm{ch}}/(N_{\mathrm{SCGB}}+N_{\mathrm{LJ}})$. The crosslinker rate $r_{\mathrm{cl}}$ was determined by $N_{\mathrm{cl}}/(N_{\mathrm{SCGB}}+N_{\mathrm{LJ}})$. The initial mesogenic orientation (IMO) direction with respect to the elongation direction was set as a logical value corresponding to three situations: vertical, parallel, and isotropic. All the LCE systems had a well-defined isotropic–nematic phase transition temperature $T_{\mathrm{NI}}$. The system temperature during elongation $T_{\mathrm{elong}}$ was set as described in Method. Table 2 presents the parameters for the system and simulation conditions described above. Note that some of the system and simulation conditions described in Method are fixed to specific values and are therefore not considered as descriptors; these are not listed in Table 2.

**Table 2:** Variables for the system and simulation conditions of the LCE molecular systems.

System conditions Variable	Type	Description	Value(s)
$N_{\mathrm{ch}}$	Integer	Number of chains	105, 111, 118, 125, 133, 175, 225, 250
$N_{\mathrm{MU}}$	Integer	Number of mesogenic units	$m_{\mathrm{ru}} n_{\mathrm{MU}} N_{\mathrm{ch}}$
$N_{\mathrm{solv}}$	Integer	Number of solvent SCGB particles	$k N_{\mathrm{MU}}$ $(k=1,2,\ldots )$
$N_{\mathrm{MU,cl}}$	Integer	Number of mesogenic units in crosslinkers	$m_{\mathrm{cl}} n_{\mathrm{MU,cl}} N_{\mathrm{ch}}$
$N_{\mathrm{SCGB}}$	Integer	Total number of SCGB particles	$N_{\mathrm{MU}}+N_{\mathrm{MU,cl}}+N_{\mathrm{solv}}$
$N_{\mathrm{LJ,ch}}$	Integer	Number of LJ units in chains	$m_{\mathrm{ru}} (n_{\mathrm{LJ,mc}}+n_{\mathrm{LJ,sc}}) N_{\mathrm{ch}}$
$N_{\mathrm{LJ,cl}}$	Integer	Number of LJ particles in crosslinkers	$m_{\mathrm{cl}} n_{\mathrm{LJ,cl}} N_{\mathrm{ch}}$
$N_{\mathrm{LJ}}$	Integer	Total number of LJ particles	$N_{\mathrm{LJ,ch}}+N_{\mathrm{LJ,cl}}$
$N_{\mathrm{cl}}$	Integer	Total number of crosslinkers	$m_{\mathrm{cl}}N_{\mathrm{ch}}$
$r_{\mathrm{ch}}$	Real	Chain rate	$N_{\mathrm{ch}}/(N_{\mathrm{SCGB}}+N_{\mathrm{LJ}})$
$r_{\mathrm{cl}}$	Real	Crosslinker rate	$N_{\mathrm{cl}}/(N_{\mathrm{SCGB}}+N_{\mathrm{LJ}})$
$T_{\mathrm{NI}}$	Real	Isotropic–nematic phase transition temperature	
**Simulation conditions**			
Variable	Type	Description	Value(s)
IMO	Logical	Initial mesogenic orientation direction with respect to the elongation direction	Null (= isotropic), True (= vertical), False (= parallel)
$T_{\mathrm{elong}}$	Real	System temperature during elongation	

**Figure 2:**
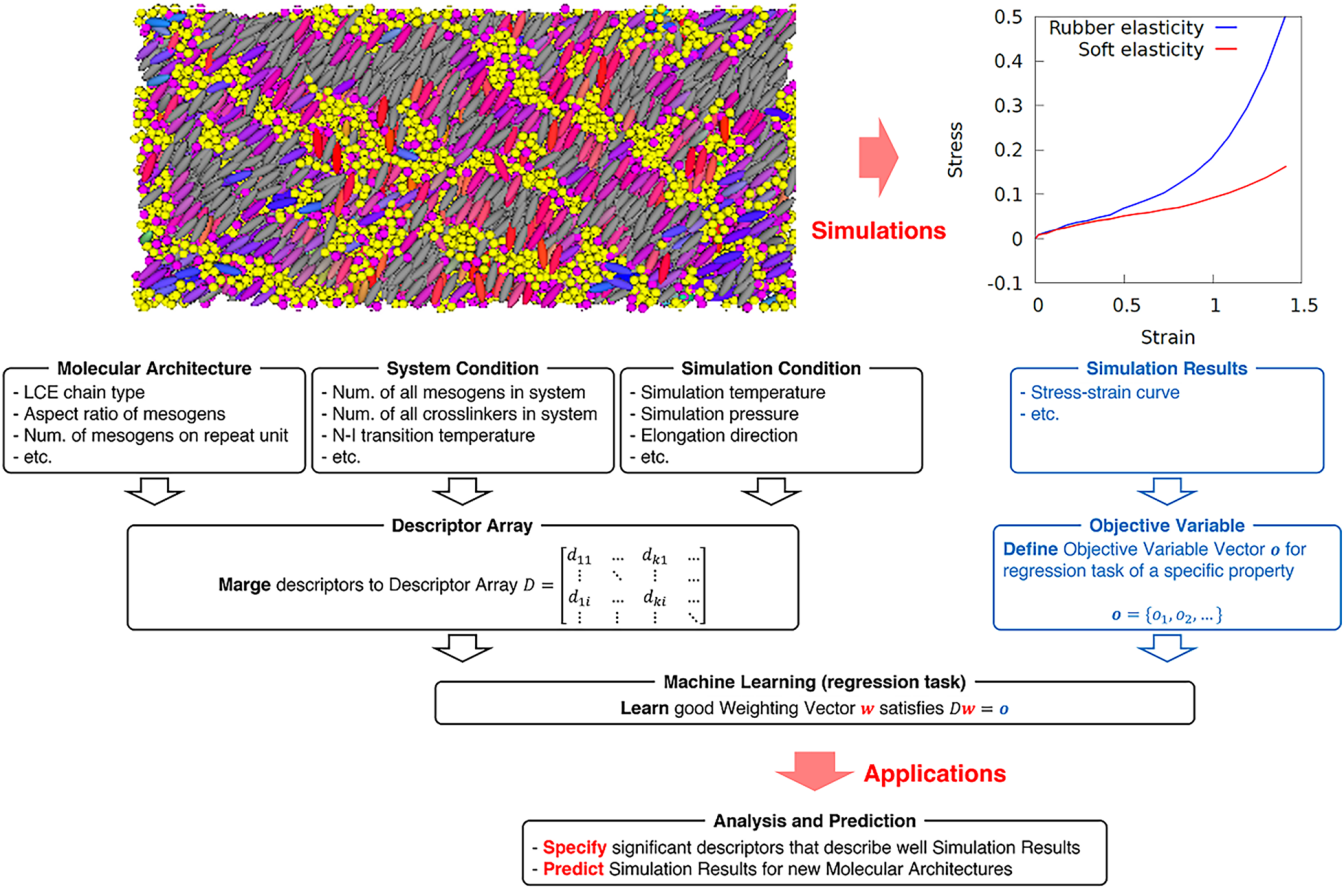
ML flow in this study.

A simple supervised ML scheme was used for the regression task, because the close relation between input microscopic descriptors and objective macroscopic properties is assumed to be obvious and there is no need to use complex ML methods that often contain implicit parameters. Figure 2 shows the ML procedure employed in this study. First, the parameters of the microscopic molecular systems were used as descriptors for the regression task of ML; that is, the molecular architecture information and the system and simulation conditions were merged into a descriptor array *D*. The 33 descriptors used in this study are listed in Table 3. $L_{\mathrm{mov}}$ was added as a descriptor to express the spacing for the mobility of mesogenic units. This is set to $L_{\mathrm{mc}}+L_{\mathrm{sc}}$ in Table 1. The descriptor $r_{\mathrm{temp}}$ expresses the relative temperature during elongation from the nematic–isotropic transition temperature. Other descriptors listed in Tables 1 and 2 are restated in Table 3 for convenience. Next, the stress–strain curve data obtained from coarse-grained MD simulations of LCE molecular systems were selected as the objective variables. Specifically, the combinations of stress and strain values that form discrete points on the curve were stored in the objective variable vector ${\varvec{o}}$. Each curve consists of 20 discrete points. The above descriptors and objective variables were stored in a database in the format shown in Fig. S1 of the Supplementary Materials. The database contains descriptors and objective variables for a total of 220 LCE molecules. Considering the bias of the stored LCE molecular architectures, 80 LCE molecules were excluded and the remaining 140 LCE molecules were selected for this study. From these 140 molecules, another 12 molecules were randomly excluded. Thus, regression analysis was applied to the data of 128 LCE molecules. The size of the descriptor array is 128 (LCE molecules) $\times$ 33 (descriptors) = 4,224, while the number of data for the stress–strain curves is 128 (LCE molecules) $\times$ 6 (curves) $\times$ 20 (points) = 15,360. Finally, the operator vector ${\varvec{w}}$ satisfying the relation $D {\varvec{w}} = {\varvec{o}}$ was estimated via ML. The term ${\varvec{w}}$ was estimated using the random forest method^66^ implemented on Scikit-learn (version 0.20.3)^67^. The random forest method was employed for the following four main reasons. (i) The method does not require data normalization and is relatively simple, with the only hyperparameters being the number of trees $n_{\mathrm{tree}}$ and the depth of tree $d_{\mathrm{tree}}$. (ii) The stress-strain curves of LCE, the objective variables, are complex discrete data, but can be handled stably by using the random forest. (iii) The random forest can quantify the importance of descriptors including design variables for LCE molecules. (iv) The benchmarks presented in a previous study using molecular information as descriptors showed that the method using decision trees performed better^68^. Note that the hyperparameters of the random forest, $n_{\mathrm{tree}}$ and $d_{\mathrm{tree}}$, were determined to be 100 and 10, respectively, after sufficient exploration by grid search with $n_{\mathrm{tree}}$ up to 1000 and $d_{\mathrm{tree}}$ up to 50. All other parameters were set to the default values of Scikit-learn. Also, the random forest method has some other useful characteristics as the ML algorithm: (i) the learning routine is simple, and thus achieves high-performance computing, (ii) the method avoids overlearning, and (iii) little or no data cleansing is needed. Note that overlearning refers to the scenario in which the learning results only fit the data used during learning and do not fit new data. The vector ${\varvec{w}}$ was checked using *k*-fold cross-validation for overlearning implemented on Scikit-learn, where *k* (set to 10 in this study) denotes the number of times cross-validation was performed.

**Table 3:** Descriptors used in the ML process. Some variables fixed to specific values are not used because they do not affect the ML results.

Descriptor	Type	Description	Value(s)
MA	Logical	Main- or side-chain type	True (= main), False (= side)
$n_{\mathrm{LJ,mc}}$	Integer	Number of LJ units in the main-chain of repeat unit	Null, 1, 2, 3
$n_{\mathrm{LJ,sc}}$	Integer	Number of LJ units in the side-chain of repeat unit	Null, 1, 2, 3
*q*	Real	Point charge on the mesogenic unit	Null, 0.3
$l_{\mathrm{MM}}$	Real	Length of the covalent bond between mesogenic units	Null, 0.15, 1, 1.2, 1.4, 1.6, 1.8, 2.0, 3.0
$l_{\mathrm{LL}}$	Real	Length of the covalent bond between LJ particles	Null, 1.0
$l_{\mathrm{LM}}$	Real	Length of the covalent bond between LJ particle and mesogenic unit	Null, 0.5
$k_0$	Real	Spring constant of the covalent bond	100.0, 500.0
CL	Logical	Presence or absence of the crosslinker point with the side chain	True (= present), False (= absent)
$m_{\mathrm{cl}}$	Integer	Number of crosslinkers in the chain	2, 3, 4, 6, 8
$n_{\mathrm{MU,cl}}$	Integer	Number of mesogenic units in the crosslinker	Null, 1
$n_{\mathrm{LJ,cl}}$	Integer	Number of LJ units in the crosslinker	Null, 1, 2, 3, 4, 5, 6
$k_{\theta }$	Real	Angle spring constant of the LJ crosslinker	Null, 10.0, 1000.0
$l_{\mathrm{MM, cl}}$	Real	Length of the covalent bond between mesogenic units in the main-chain and mesogenic units in the crosslinker	Null, 0.0, 0.15, 0.5, 1.0
$l_{\mathrm{LL, cl}}$	Real	Length of the covalent bond between LJ particles in the main-chain and crosslinker	Null, 1.0
$l_{\mathrm{LM, cl}}$	Real	Length of the covalent bond between LJ particle in the main-chain and mesogenic unit in the crosslinker	Null, 0.15, 0.5
$r_{\mathrm{asp}}$	Real	Aspect ratio of the SCGB particle	$\sigma _{\mathrm{e}}/\sigma _{\mathrm{s}}$
$L_{\mathrm{mov}}$	Real	Spacing for the mobility of mesogenic units	$L_{\mathrm{mc}}+L_{\mathrm{sc}}$
$N_{\mathrm{ch}}$	Integer	Number of chains	105, 111, 118, 125, 133, 175, 225, 250
$N_{\mathrm{MU}}$	Integer	Number of mesogenic units	$m_{\mathrm{ru}} n_{\mathrm{MU}} N_{\mathrm{ch}}$
$N_{\mathrm{solv}}$	Integer	Number of solvent SCGB particles	$k N_{\mathrm{MU}}$ $(k=1,2,\ldots )$
$N_{\mathrm{MU,cl}}$	Integer	Number of mesogenic units in crosslinkers	$m_{\mathrm{cl}} n_{\mathrm{MU,cl}} N_{\mathrm{ch}}$
$N_{\mathrm{SCGB}}$	Integer	Total number of SCGB particles	$N_{\mathrm{MU}}+N_{\mathrm{MU,cl}}+N_{\mathrm{solv}}$
$N_{\mathrm{LJ,ch}}$	Integer	Number of LJ units in chains	$m_{\mathrm{ru}} (n_{\mathrm{LJ,mc}}+n_{\mathrm{LJ,sc}}) N_{\mathrm{ch}}$
$N_{\mathrm{LJ,cl}}$	Integer	Number of LJ particles in crosslinkers	$m_{\mathrm{cl}} n_{\mathrm{LJ,cl}} N_{\mathrm{ch}}$
$N_{\mathrm{LJ}}$	Integer	Total number of LJ particles	$N_{\mathrm{LJ,ch}}+N_{\mathrm{LJ,cl}}$
$N_{\mathrm{cl}}$	Integer	Total number of crosslinkers	$m_{\mathrm{cl}}N_{\mathrm{ch}}$
$r_{\mathrm{ch}}$	Real	Chain rate	$N_{\mathrm{ch}}/(N_{\mathrm{SCGB}}+N_{\mathrm{LJ}})$
$r_{\mathrm{cl}}$	Real	Crosslinker rate	$N_{\mathrm{cl}}/(N_{\mathrm{SCGB}}+N_{\mathrm{LJ}})$
$T_{\mathrm{NI}}$	Real	Isotropic–nematic phase transition temperature	
IMO	Logical	Initial mesogenic orientation direction with respect to the elongation direction	Null (= isotropic), True (= vertical), False (= parallel)
$T_{\mathrm{elong}}$	Real	System temperature during elongation	
$r_{\mathrm{temp}}$	Real	Temperature rate	$T_{\mathrm{elong}}/T_{\mathrm{NI}}$

**Figure 3:**
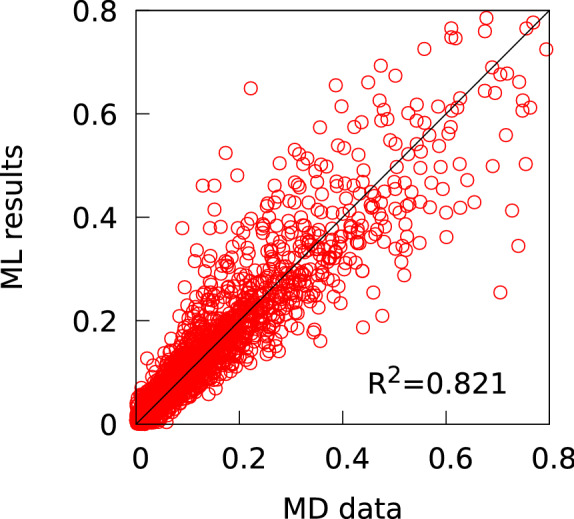
Regression curves of stress corresponding to various strain values on the LCE stress–strain curves.

Figure 3 shows the regression curves of stress corresponding to various strain values on the LCE stress–strain curves. The R-squared value is 0.821, which indicates a strong correlation between the database values and ML results. The mean absolute error (MAE) was 0.065. Table 4 presents a subset of the descriptors in descending order of importance score derived from the random forest decision tree. The most significant component is IMO, which contributes 26 % of the importance score. This indicates that the relation between the initial mesogen orientation and elongation direction is a key parameter in the elasticity of LCEs. Experiments have achieved a rich variety of LCE actuation by precisely adjusting the initial orientation direction of mesogens relative to the deformation direction^5,15,23,69–72^. The conceptual quantities $r_{\mathrm{cl}}$ and $r_{\mathrm{ch}}$, which reflect the crosslink density and polymer chain density, respectively, are important parameters in LCE molecular systems. While a previous study reported that the crosslink density systematically controls the trade-off relationship between the stress and strain range during soft elasticity, it has also been revealed that modulating the polymer chain density may increase both the stress and strain range during soft elasticity, moving beyond the trade-off relationship^35^. The results show that $r_{\mathrm{cl}}$ and $r_{\mathrm{ch}}$ account for 21 % and 17 % of the importance score, respectively. The simulation condition $T_{\mathrm{elong}}$ constitutes 8 % of the importance score. The design variables $n_{\mathrm{LJ,cl}}$, $L_{\mathrm{mov}}$, MA, and $l_{\mathrm{MM}}$ make up 6 %, 5 %, 4 %, and 4 % of the importance score, respectively. $T_{\mathrm{NI}}$ is a physical property of the LCE system, and contributes 6 % of the importance score. The nine descriptors stated above constitute 97 % of the importance score. Note that the other descriptors contribute less than 1 % each to the importance score. Because $n_{\mathrm{LJ,cl}}$, $L_{\mathrm{mov}}$, MA, and $l_{\mathrm{MM}}$ are molecular architecture descriptors, $r_{\mathrm{cl}}$, $r_{\mathrm{ch}}$, and $T_{\mathrm{NI}}$ are system condition descriptors, and IMO and $T_{\mathrm{elong}}$ are simulation condition descriptors, we can determine that the molecular structure, system conditions, and simulation conditions make contributions of approximately 19 %, 44 %, and 34 %, respectively, to the elasticity of LCEs. The regression analysis shows that the design variables of the LCE molecules contribute to about one-fifth of the elasticity of LCE systems. Among the design variables for the LCE molecular architecture, it is worth noting that $L_{\mathrm{mov}}$, MA, and $l_{\mathrm{MM}}$ are inseparable from the mesogenic rotation mechanism. Our previous study revealed differences in the mechanism of soft elasticity between main-chain and side-chain LCEs, which are explained by differences in the mesogenic rotation mechanism^34^. The regression analysis seems to capture this feature.

Overall, the regression analysis succeeded in capturing the following three factors that are important in the soft elasticity mechanism: (i) the relationship between initial orientation direction and deformation direction, (ii) the influence of the crosslink density and polymer chain density, and (iii) the difference between main-chain and side-chain mesogenic rotation mechanisms.

**Table 4:** Importance score of descriptors for stress–strain curves derived from the decision tree (descending order).

Descriptor	Importance score (%)
IMO	26
$r_{\mathrm{cl}}$	21
$r_{\mathrm{ch}}$	17
$T_{\mathrm{elong}}$	8
$n_{\mathrm{LJ,cl}}$	6
$T_{\mathrm{NI}}$	6
$L_{\mathrm{mov}}$	5
MA	4
$l_{\mathrm{MM}}$	4

### Prediction of elasticity of LCEs

**Figure 4:**
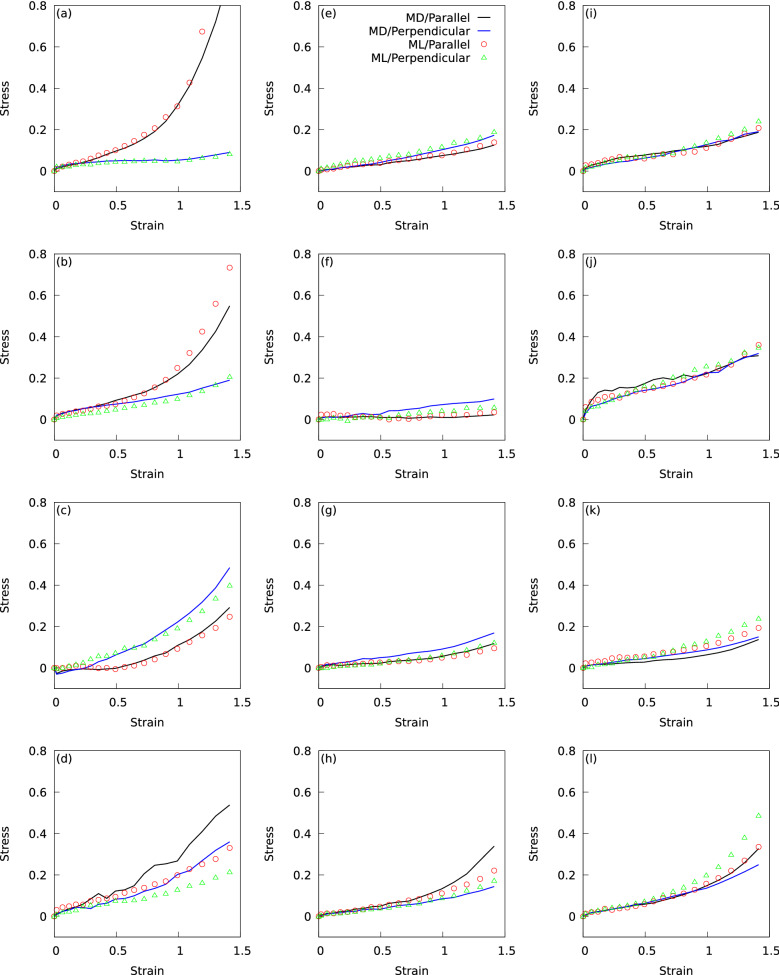
Comparison of the ML predictions and MD results for the stress–strain curves.

The surrogate model constructed by the regression analysis should be able to predict the elasticity of LCE molecular systems that were not used in the regression analysis. Therefore, we applied the surrogate model to 12 LCE molecular systems that were not used in the regression analysis to test its ability to predict stress–strain curves. The predictions were formulated in two simple steps. First, descriptors for the 12 LCE molecules were extracted from the database to generate a new *D*. The descriptors for the 12 LCE molecules are shown in the Supplementary Data. Second, the stress–strain curves of the 12 LCE molecules were predicted by computing $D{\varvec{w}}$. Recall that ${\varvec{w}}$ is the surrogate model obtained by applying the ML scheme to the data of 128 LCE molecules. Figure 4 compares the ML predictions and MD results for the stress–strain curves. Panels (a)–(l) are the results for LCE molecular systems with different molecular architectures from each other. The stress–strain curves were predicted by the ML scheme for elongations both parallel and perpendicular to the initial mesogen orientation. In panels (a)–(d), the anisotropy of the LCE deformation caused by the onset of soft elasticity is obvious; in panels (e)–(h), the anisotropy is weak because the onset of soft elasticity is not obvious; in panels (i)–(l), there is no anisotropy because of the absence of soft elasticity. When the anisotropy of the LCE deformation is clear, the ML predictions follow the MD results well, i.e., panels (a)–(c). In panel (d), the direction of elongation in which soft elasticity develops has been predicted. In the absence of LCE deformation anisotropy, the ML predictions in panels (i)–(k) follow the MD results closely. In panel (l), as the strain increases, a discrepancy appears between the MD results and ML predictions for the elongation perpendicular to the initial mesogen orientation. Clarifying the cause of this discrepancy is not easy because the top descriptors (Table 4) influence the stress-strain curves in a complicated and combined manner, and factors other than the top descriptors could also have some effect. Future development of surrogate model that captures stress anisotropy more accurately may provide clues for improving the prediction capability. For weakly anisotropic LCE deformations, panel (e) shows that the ML predictions track the MD results well. In panels (f)–(h), the surrogate model does not predict the slight anisotropy observed in the MD results, but does predict the relationship between large and small stresses in different elongation directions.

**Figure 5:**
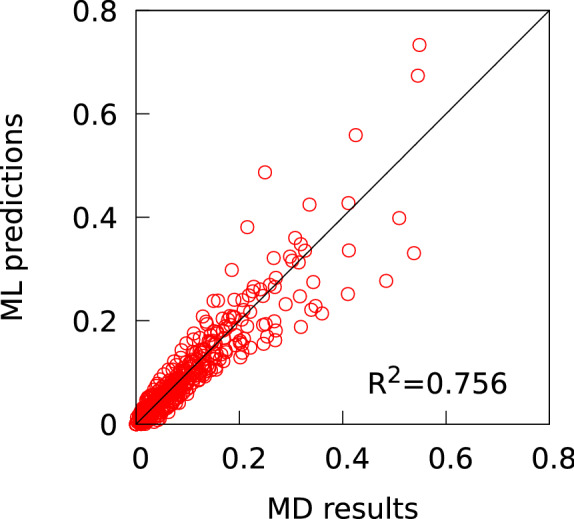
Regression predictions against the MD calculated values for the stresses corresponding to various strain values in the stress-strain curves for the 12 LCEs.

Figure 5 plots the regression predictions against the MD calculated values for the stresses corresponding to various strain values in the stress-strain curves for the 12 LCEs (Fig. 4). The R-squared value was 0.756 and the MAE was 0.023, indicating that the accuracy of ML prediction for the MD results is comparable to the regression accuracy shown in Fig. 3.

Overall, the surrogate model has a strong ability to predict stress–strain curves for unknown LCE molecules. In particular, in the case of strong or little anisotropy of LCE deformations (panels (a)-(d) and (i)-(l)), the model predicts the curve quantitatively in 6/8 cases, and predicts the stress relationship between large and small stresses caused by differences in the elongation direction in 7/8 cases. Considering that we are mainly interested in the case of strong anisotropy of LCE deformations in the search for LCE materials, the surrogate model provides a useful tool in the selection of LCE molecules.

## Discussion

In this study, we attempted to identify the microscopic characteristics governing the macroscopic deformation of LCEs using a QSPR approach refined by supervised ML. From a database containing the results of coarse-grained MD simulations of LCEs with various molecular structures, the design variables of the molecular architecture, the molecular system conditions, and the simulation conditions for 140 different LCE molecules were extracted as explanatory variables, while the stress–strain curves resulting from the simulations were extracted as objective variables. First, regression analysis was performed using the explanatory and objective variables for 128 randomly selected LCE molecules to identify the design variables of the molecular architecture that govern the macroscopic deformation of the LCEs. The key descriptors revealed by the regression analysis suggest that the surrogate model obtained is capable of representing three important elements in the soft elasticity mechanism: the relationship between initial orientation direction and deformation direction^5,15,23,69–72^, the influence of the crosslink density and polymer chain density^35^, and the difference between the main-chain and side-chain mesogenic rotation mechanisms^34^. Next, to verify the predictive performance of the surrogate model obtained by regression analysis, the stress–strain curves were predicted using ML results for 12 LCE molecules that were excluded from the regression. The surrogate model was shown to have a good ability to predict stress–strain curves for unknown LCE molecules. In particular, for cases with strong or little anisotropy in their LCE deformations, the curves were quantitatively predicted in 6/8 cases and the relationship between large and small stresses caused by differences in the elongation direction was predicted in 7/8 cases. Future research will attempt to incorporate more sophisticated ML methods for the case of weak anisotropy of the LCE deformations. However, the surrogate model established in this study already has the potential to select LCE molecules for designing materials that exhibit strong anisotropy of LCE deformations. Of course, the parameters that determine the physical properties of LCEs range from the primary structure of mesogens and polymer chains to higher-order structures such as mesogen orientation, cross-linking networks, and mixing distributions. Therefore, the macroscopic properties of LCEs may not be fully explained by molecular architecture alone. This point is expected to be filled by simulations, experiments, and informatics on a more macroscopic scale, or by fusion studies of these.

## Methods

Uniaxial elongation simulations were performed under a single system condition and simulation condition for each of the 220 different molecular architectures of LCE. Three initial LCE molecular systems were prepared, each elongated in the two directions described below, resulting in six stress–strain curves per LCE molecule. The nematic-like initial structures of the LCE systems were carefully prepared. All LCE systems with different molecular architectures were first formed as isotropic structures and then gradually cooled. The Onsager order parameter *S* was monitored during the cooling simulations, and the temperature dependence of the order parameter was recorded to detect $T_{\mathrm{NI}}$ of the LCEs. For the initial elongation conditions, three structures were prepared under the temperature condition that *S*=0.7 (nematic) for each LCE system^34,35^. As the initial structures for the simulation experiments, the nematic-like structures were carefully prepared so that the mesogen orientation followed one of the rectangular cells. $T_{\mathrm{elong}}$ was set to be the same as that for the initial conditions. The MD simulations of uniaxial elongation were performed using COGNAC^73^. The Verlet velocity algorithm with a time step of $\Delta t = 0.002 \, \sigma _{0} (m_0 / \varepsilon _0)^{1/2}$ was used for time evolution under Newton’s equations of motion, where $\sigma _0$ is the characteristic van der Waals diameter, $\varepsilon _0$ is the characteristic interaction strength, and $m_0$ is the mass of the SCGB particles. In this study, $\sigma _0$, $\varepsilon _0$, and $m_0$ were set to unity, and $\sigma _{\mathrm{s}}$ was set to $\sigma _0$. Uniaxial elongation was performed for a constant number of particles, constant temperature, and two-dimensional constant pressure ensemble. The pressure *P* for the two orthogonal directions perpendicular to the elongation direction was controlled at a constant value of $10.0 \, \varepsilon _{0} / \sigma _{0}^{3}$. Elongation was emulated by deforming the cell with a deformation rate of $\dot{ \varepsilon } = 6\times 10 ^{-5} \, (\varepsilon _{0} / m_{0})^{1/2} / \sigma _{0}$ in the elongation direction. The stress–strain curves were calculated for two different elongation directions for each of the three different initial molecular structures, i.e., elongation parallel or perpendicular to the initial mesogen orientation.

## Supplementary Information

Supplementary Figure S1.

Supplementary Information.

## Data Availability

The datasets used and/or analysed during the current study available from the corresponding author on reasonable request.
