# Supplementary material for: Regression analysis for predicting the elasticity of liquid crystal elastomers
Source: Sci Rep. 2022 Nov 17;12:19788. doi: 10.1038/s41598-022-23897-0 (PMC9672114; doi:10.1038/s41598-022-23897-0)
Supplement: Supplementary file 1 — Supplementary Figure S1. [file 41598_2022_23897_MOESM1_ESM.pdf]

Supplementary Materials for

Regression analysis for predicting the elasticity of liquid crystal elastomers

Hideo Doi,[1,+] Kazuaki Z. Takahashi,[1,+,*] Haruka Yasuoka,[2,3,+]
Jun-ichi Fukuda,[4] Takeshi Aoyagi[1]

[1] National Institute of Advanced Industrial Science and Technology (AIST),
Research Center for Computational Design of Advanced Functional Materials,
Central 2, 1-1-1 Umezono, Tsukuba, Ibaraki 305-8568, Japan
[2] Research Association of High-Throughput Design and Development
for Advanced Functional Materials,
Central 2, 1-1-1 Umezono, Tsukuba, Ibaraki 305-8568, Japan
[3] Panasonic Corporation,
3-1-1 Yagumo-naka-machi, Moriguchi City, Osaka 570-8501, Japan
[4] Kyushu University,
Department of Physics, Faculty of Science,
744 Motooka, Nishi-ku, Fukuoka, Fukuoka 819-0395, Japan

*To whom correspondence should be addressed; E-mail: kazu.takahashi@aist.go.jp
+These authors contributed equally to this work

This PDF file includes:

Figure S1

Other Supplementary Materials for this manuscript includes the following:

Supplementary Data

(a) Descriptors

id	data_filename	init_direct	main1:side0	nGBunit	nLJmainunit	nLJsideunit	nRepeat	nChain	GBbondL	nGBcross	nLJcross	CrLn_Dens	CrossbondL	shareSite	nGBnetwork	nGBsolv	nGBtotal	bond_k
2	matome/1LJcross_m1s0/data2/T38_DRz6em5_NPT_z_out.csv	2	0	1	1	0	30	125	-1	0	1	2	1	0	3750	3750	7500	500
3	matome/1LJcross_m1s0/data3/T38_DRz6em5_NPT_z_out.csv	2	0	1	1	0	30	125	-1	0	1	2	1	0	3750	3750	7500	500
4	matome/1LJcross_m1s0/data4/T38_DRz6em5_NPT_z_out.csv	2	0	1	1	0	30	125	-1	0	1	2	1	0	3750	3750	7500	500
5	matome/1LJcross_m1s0/data1/T38_DRz6em5_NPT_x_out.csv	1	0	1	1	0	30	125	-1	0	1	2	1	0	3750	3750	7500	500
10	matome/1LJcross_m1s1/data2/T36_DRz6em5_NPT_z_out.csv	2	0	1	1	1	30	125	-1	0	1	2	1	0	3750	3750	7500	500
11	matome/1LJcross_m1s1/data3/T36_DRz6em5_NPT_z_out.csv	2	0	1	1	1	30	125	-1	0	1	2	1	0	3750	3750	7500	500
12	matome/1LJcross_m1s1/data4/T36_DRz6em5_NPT_z_out.csv	2	0	1	1	1	30	125	-1	0	1	2	1	0	3750	3750	7500	500
13	matome/1LJcross_m1s1/data1/T36_DRz6em5_NPT_x_out.csv	1	0	1	1	1	30	125	-1	0	1	2	1	0	3750	3750	7500	500
14	matome/1LJcross_m1s1/data2/T36_DRz6em5_NPT_x_out.csv	1	0	1	1	1	30	125	-1	0	1	2	1	0	3750	3750	7500	500
15	matome/1LJcross_m1s1/data3/T36_DRz6em5_NPT_x_out.csv	1	0	1	1	1	30	125	-1	0	1	2	1	0	3750	3750	7500	500
16	matome/1LJcross_m1s1/data4/T36_DRz6em5_NPT_x_out.csv	1	0	1	1	1	30	125	-1	0	1	2	1	0	3750	3750	7500	500
17	matome/1LJcross_m1s2/data1/T34_DRz6em5_NPT_z_out.csv	2	0	1	1	2	30	125	-1	0	1	2	1	0	3750	3750	7500	500
18	matome/1LJcross_m1s2/data2/T34_DRz6em5_NPT_z_out.csv	2	0	1	1	2	30	125	-1	0	1	2	1	0	3750	3750	7500	500
19	matome/1LJcross_m1s2/data3/T34_DRz6em5_NPT_z_out.csv	2	0	1	1	2	30	125	-1	0	1	2	1	0	3750	3750	7500	500
20	matome/1LJcross_m1s2/data4/T34_DRz6em5_NPT_z_out.csv	2	0	1	1	2	30	125	-1	0	1	2	1	0	3750	3750	7500	500
21	matome/1LJcross_m1s2/data1/T34_DRz6em5_NPT_x_out.csv	1	0	1	1	2	30	125	-1	0	1	2	1	0	3750	3750	7500	500
22	matome/1LJcross_m1s2/data2/T34_DRz6em5_NPT_x_out.csv	1	0	1	1	2	30	125	-1	0	1	2	1	0	3750	3750	7500	500
23	matome/1LJcross_m1s2/data3/T34_DRz6em5_NPT_x_out.csv	1	0	1	1	2	30	125	-1	0	1	2	1	0	3750	3750	7500	500
24	matome/1LJcross_m1s2/data4/T34_DRz6em5_NPT_x_out.csv	1	0	1	1	2	30	125	-1	0	1	2	1	0	3750	3750	7500	500
25	matome/1LJcross_m2s0/data1/T34_DRz6em5_NPT_z_out.csv	2	0	1	2	0	30	125	-1	0	1	2	1	0	3750	3750	7500	500

(b) Objective variables

s10	s11	s12	s13	s14	s15	s16	s17	s18	s19
0.14443799678539998	0.15147903700899998	0.1481167672011	0.16129220806269998	0.1536322601724	0.15870048209049997	0.15310599894789997	0.16212294600649998	0.1726268559728	0.17584481763870002
0.07486330631371	0.08165224208231	0.06070876768411	0.06391428209046	0.08304039539773	0.08197675108836999	0.08505847969805999	0.08773255982817	0.09702345161980999	0.09725377423746001
0.12404617392910003	0.1181590866818	0.121469912384	0.12699052894829999	0.13488429542269997	0.1434950742482	0.1376882549195	0.1461594636346	0.15092362739360002	0.15856521216039998
0.0963144608563	0.10317845566891999	0.1218862024562	0.1387199239217	0.1431946370601	0.14432034277360004	0.13640033251349998	0.16433035207529997	0.1888361868176	0.2178376679579
0.035228983101210004	0.040856124771699996	0.04930925501707	0.04548937010361	0.06031920773215001	0.06037539114803	0.05252169519365	0.061090809708310004	0.057566991665239986	0.07566885970611
0.04822205496920999	0.05884527442945	0.07253925999819999	0.06639952077868	0.06516420602274	0.06927876542669001	0.0913641842531	0.10530012668122998	0.10606162339839001	0.11214287758563998
0.08766348915008	0.0919131130147	0.10264917733661001	0.11495095294680002	0.1159071615443	0.11995529362629997	0.12572339159239998	0.12947522246	0.14510950916010001	0.15141213417649999
0.10930998483348	0.10947904104773001	0.13563481006100003	0.140944591956	0.1436174101909	0.1410100653165	0.16088884984159998	0.1664800079838	0.1797382878216	0.1918125976764
0.08546237557171998	0.10092353081627999	0.10414019197757	0.11584595766250001	0.12307851258400002	0.12602134868690001	0.1454894793362	0.16035823579509997	0.18471565608989998	0.18765668083040002
0.1288716229313	0.14153197055330002	0.1450326948255	0.1527786682649	0.1604372293547	0.1688828009603	0.1666735583248	0.1858833607359	0.2118870162466	0.2385591559516
0.08969866205314	0.09606323057492001	0.10613813745823	0.1191263440156	0.10793947881132	0.13227636286439998	0.12373262629840003	0.130056960917	0.13957619212869998	0.15031180364650001
0.06141766537675999	0.06488619692459	0.07960488342149	0.07769223831592	0.067546840467	0.061967978310099994	0.07841330316923999	0.07273938391780001	0.09054428782205999	0.09343350947966
0.053838163716710005	0.04495575446005	0.05169239391403	0.06351058877049001	0.05190386059699	0.06673290833477	0.08072629529991002	0.08105905622859	0.08718718923979	0.08470388808892

Figure S1: Database image. (a) descriptors and (b) objective variables are stored.
